# National control laboratory independent lot testing of COVID-19 vaccines: the UK experience

**DOI:** 10.1038/s41541-021-00368-7

**Published:** 2021-08-12

**Authors:** Nicola J. Rose, Paul Stickings, Silke Schepelmann, Marc J. A. Bailey, Chris Burns

**Affiliations:** 1 The National Institute for Biological Standards and Control, a Centre of the Medicines and Healthcare products Regulatory Agency, Division of Virology, Hertfordshire, United Kingdom; 2 The National Institute for Biological Standards and Control, a Centre of the Medicines and Healthcare products Regulatory Agency, Division of Bacteriology, Hertfordshire, United Kingdom; 3 The National Institute for Biological Standards and Control, a Centre of the Medicines and Healthcare products Regulatory Agency, Hertfordshire, United Kingdom; 4 The National Institute for Biological Standards and Control, a Centre of the Medicines and Healthcare products Regulatory Agency, Division of Biotherapeutics, Hertfordshire, United Kingdom

**Keywords:** Viral infection, Public health

## Abstract

The past 18 months have seen an unprecedented approach to vaccine development in the global effort against the COVID-19 pandemic. The process from discovery research, through clinical trials and regulatory approval often takes more than 10 years. However, the critical need to expedite vaccine availability in the pandemic has meant that new approaches to development, manufacturing, and regulation have been required: this has necessitated many stages of product development, clinical trials, and manufacturing to be undertaken in parallel at a global level. Through the development of these innovative products, the world has the best chance of finding individual, or combinations of, vaccines that will provide adequate protection for the world’s population. Despite the huge scientific and regulatory achievements and significant investment to accelerate vaccine availability, it is essential that safety measures are not compromised. Here we focus on the post regulatory approval testing by independent laboratories that provides an additional assurance of the safety and quality of a product, with an emphasis on the UK experience through the National Institute for Biological Standards and Control (NIBSC), an expert centre of the UK’s Medicines and Healthcare products Regulatory Agency (MHRA).

## Introduction

Biological medicines, including vaccines, are complex products requiring specialised analytical tests to ensure appropriate quality and safety. Unlike the testing of chemical drugs, physio-chemical methods are not always applicable. Vaccines have an inherent variability due to the product itself as well as the nature of the manufacturing process and the tests used to evaluate the material. As an example of the latter, biological safety and potency profiling will typically include biological assays requiring cells and microorganisms.

Both approved and candidate COVID-19 vaccines combine platforms that have been used widely before, such as inactivated virus and virus subunit-based vaccines, with technologies more recent to the vaccine field, including viral vectors and mRNA and self-amplifying (sa)RNA (reviewed in^1,2^). These different vaccine types require a range of specific biological and physio-chemical methods for quality and safety assessment.

Production runs (often termed batches or lots) of final formulated vaccine can comprise hundreds of thousands of individual doses, with the result that the safety risks arising from, for example, adventitious agents, residual toxins, or residual active virus, being present in even a single batch, could be significant and the effects widespread. Likewise, a sub- or supra-potent vaccine may have an impact on protection rates or adverse reactions at an individual and population level, the evidence of which may be noticeable sooner in a pandemic than in a routine childhood immunisation programme, for example, due to greater numbers of people receiving vaccines in a relatively short period of time.

### The role of independent control testing by a National Control Laboratory (NCL)

Vaccines undergo rigorous regulatory approval procedures which include an obligation for compliance with good manufacturing practice (GMP). Vaccine manufacturers are also obliged to undertake a wide range of quality and safety tests of the components used in manufacturing, and the final formulated vaccine product, as a condition of their regulatory approval for its use, under the umbrella of Chemistry, Manufacturing, and Control (CMC) as reviewed by Sanyal and colleagues^3^. In addition to this testing by manufacturers, independent National Control Laboratories perform their own impartial assessments of vaccine batches as an additional step to control the safety and efficacy of these sensitive products prior to marketing. This process is variously termed Lot Release (World Health Organization, WHO^4^), Batch Release, Official Control Authority Batch Release (European Union; European Directorate for the Quality of Medicines, EDQM^5^), and Independent Control Testing (NIBSC^6^).

In the UK, independent batch release testing of biological materials dates back to the Therapeutic Substances Act, 1925, thus the testing performed today is underpinned by almost a century of experience and knowledge. The National Institute for Biological Standards and Control was formally established in 1976 and has played a role in safeguarding the quality of biological medicines for almost 50 years.

This activity is a critical part of the regulatory pathway and adds value in a number of ways.

- It provides reassurance in the critical quality attributes of not only the drug product but also the component parts of a particular batch.
- It acts as a verification of the validity and accuracy of the same tests performed by the manufacturer. Since the evaluations are undertaken on vaccines from all manufacturers, there is no bias towards any one product.
- The manufacturing consistency of a product can be monitored, since meaningful deviation from acceptable criteria from one batch to the next can be measured.
- Vaccines are typically given to individuals who are healthy with respect to the disease against which they are being vaccinated. Any medicine, biological or chemical may have associated side effects that can lead individuals to decline their use. In the COVID-19 era, the public is faced with novel vaccines for a new disease. An independent assessment of critical quality attributes of these vaccines may go some way to minimising vaccine hesitancy.
- The knowledge and experience gained from independent batch testing mean that there is extensive technical expertise that can support other parts of the regulatory pathway (for example, dossier assessment, inspections) and investigations in the event of a public health concern or issue with a particular product.

### Analytical approaches to independent control testing

Independent control testing of vaccines usually involves one of the following: (a) a suite of laboratory-based tests and review of the manufacturer’s data presented in documentation often referred to as the Lot Release Protocol, (b) review of the Lot Release Protocol only, or (c) reliance on certification from another competent authority.

Under the current pandemic, the conditions for approval of COVID-19 vaccines, including emergency use authorisation differ between regulatory authorities, and thus the requirement for independent testing may also vary. Where countries elect to undertake their own independent testing and review of manufacturer data, this requires an infrastructure to manage in vitro and possibly also in vivo tests, ready access to specialist equipment, scientific and technical expertise, all supported by an appropriate quality management system (QMS). The NCL, if satisfied that a batch meets the specified criteria outlined in the product’s licensing approval, may apply a certificate indicating that the batch can be marketed in the territory covered by its authority.

Establishing the appropriate tests for a vaccine requires close cooperation between the manufacturer and the NCL. Ordinarily, manufacturers are advised to start working with their nominated control laboratory around 1 year in advance of a product being licensed, in order to ensure the ‘technical transfer’ and verification of methodologies specific to the product. Guidelines or monographs may be developed in this time to guide NCLs in the product parameters to be tested: to keep pace with the COVID-19 vaccine regulatory framework, these may exist for a time as interim documents. Some compendial tests that are detailed in Pharmacopoeia tend to be more straightforward and require less establishment and validation time, such as the physical appearance, colour, and clarity of the finished product.

With the COVID-19 pandemic, technical transfer timelines have had to be compressed, reflecting new regulatory approaches to essential vaccines. To avoid any delays to vaccine deployment, the rapid implementation of relevant tests required significant collaboration in a period when the manufacturers might still be verifying and validating the tests themselves. At NIBSC, and likely other NCLs, early engagement with manufacturers to select the necessary tests, to identify the appropriate in-house scientific and technical expertise, and ensure availability of qualified, calibrated specialist equipment, was crucial to meet the timeframes necessary for timely market access. A flexible workforce is essential to allow the appropriate deployment of staff, utilising their combination of technical, scientific, and regulatory skills. Critical to the robust, effective, and traceable transfer of methods and subsequent testing is the availability of experienced staff familiar with the quality management system underpinning independent control testing. This allowed the timely establishment of the relevant tests for a range of vaccines, including those with regulatory approval and others in different stages of the approval process.

The types of tests typically selected for lot release testing by the NCL focus on those that add value to the assurance of quality and safety of the product^7^. For the current range of COVID-19 vaccines, these laboratory-based tests are performed on the final drug product and usually include an evaluation of potency, purity, product identity, and RNA integrity. The current test regime employed by NIBSC for vaccines approved for use in the UK (and elsewhere by non-UK authorities) does not require the use of animal-based tests.

Potency assays quantify the biologically active material, with traceability to data from clinical trials through the authorisation process. Infectivity assays appropriate for a range of vaccines including viral-vectored, RNA, and protein subunit vaccines have been established and implemented at NIBSC: these require infection of cells in a multi-well format and measurement of antigen expression after a period of incubation, or enzyme-linked immunosorbent assay (ELISA). Similarly, separation and standard RNA quantification methods measure key properties of RNA, such as integrity and encapsulation for those vaccines based on this technology. The product identity can be measured variously by quantitative RT-PCR using product-specific primers and probes, western blot with antigen-specific antibody, or nucleic acid sequencing, which does not need to be next-generation sequencing if for confirmatory purposes. In some cases, the identity test can be incorporated into the potency assay, such as a product-specific PCR readout of a cell-based potency assay. When establishing tests there may be components that are defined as critical, or non-interchangeable, such as specialised equipment. The NCL may also identify assay reagents that will require validation for their fitness for use prior to establishing a robust and reproducible assay. Biological materials such as foetal calf / bovine serum, antibodies, and cell banks, are frequently validated in the NIBSC laboratories: a combination that might be optimal for one product may not be so for another thus early initiation of technical transfer of methods is recommended to maximise testing readiness. It is essential to apply the appropriate reference materials and/or system suitability controls to ensure the analyst knows when an assay does not meet validity criteria or whether a product has not met the agreed specifications: these are frequently made available by the manufacturer.

The ability to draw on experience and procedures gained from decades of operating an NCL with a staff experienced in a wide range of viral and bacterial vaccines and immunological medicinal products was invaluable in enabling NIBSC to respond quickly and effectively to establishing these analytical tests. Consequently, NIBSC was in a position to certificate batches of those vaccines granted the earliest approval, including Pfizer/BioNTech mRNA vaccine and the AstraZeneca adenovirus-vectored vaccine in December 2020^8,9^.

In the current crisis, NIBSC has encouraged manufacturers to adopt the process whereby their testing and that at the NCL are performed in parallel. In this way, the NCL testing can typically be completed ahead of the manufacturer’s longer-duration tests and thus final certification of compliant batches provides no barrier to vaccine deployment.

It is recognised that investment in complex analytical techniques such as next-generation sequencing, fragment analysis capillary electrophoresis, and ultra-performance liquid chromatography, alongside cell-based potency assays and serological tests could be prohibitive for some laboratories. In the longer term, the development of tests that can be applied to more than one type of product might allow for greater accessibility by a wider range of laboratories and this should be the focus of applied research and development programmes across the networks of NCLs. However, the possibility of an NCL recognising the results of testing or certificates from another laboratory may be a useful approach.

### Review of the manufacturer’s test data

The manufacturer’s data can be presented variously in documentation such as a Lot Release Protocol (LRP) or a Summary Production Protocol (WHO terminology). For the purpose of this Perspective, we have elected to use LRP to describe the document which contains both product and batch-specific information. The independent assessment of the LRP allows the NCL to confirm details of the manufacturer, as well as those of the product. The latter includes information on the final drug product batch (size, identification, number of containers to be released, date of manufacture, and date of expiry), its traceability to the component parts (drug substance, adjuvants, formulation buffers, etc) underpinned by a genealogy of the batch identifying the sites of manufacture and nature of critical starting materials such as seed strains and cell banks. Results of all manufacturer’s tests performed on any of the above components, alongside the specifications as set out in the authorisation, are provided, and approved by a nominated company individual, a Qualified Person. The NCL checks that the manufacturer’s data meet the authorised product’s specifications through recording the outcome of the document review process.

NCLs perform laboratory-based testing and LRP review within the QMS. This serves to ensure reliability and traceability of data through a multitude of mechanisms including, but not limited to, staff training and competency, document control, data management and trending, equipment management, out-of-specification investigation, and corrective and preventative action^7^. At NIBSC, multiple product-specific plans were established for the COVID-19 vaccines using a long-established quality framework. NIBSC has accreditation to ISO/IEC 17025:2017 from the UK’s national accreditation body (the United Kingdom Accreditation Service; UKAS) for the majority of its batch testing. Those tests not currently accredited are ISO 17025 compliant for the key technical requirements and remain subject to audit.

### Performance of manufacturing

The COVID-19 vaccines are such new products that the availability of formally established biological measurement standards or reference materials associated with them is limited. As a WHO Collaborating Centre (WHO CC) for biological standardisation, NIBSC, supported by other WHO CCs, global laboratories, and the Coalition for Epidemic Preparedness Innovations (CEPI), has developed International Standards for SARS-CoV-2 RNA and anti-SARS-CoV-2 antibody to support manufacturers with the development and evaluation of COVID-19 vaccines through assay harmonisation^10^. However, whereas WHO International Standards exist for viral vaccines such as polio, rabies and yellow fever, independently established reference materials relevant to the quality control are not available currently for the different COVID-19 vaccines. While this in itself does not hinder the NCL testing of new vaccines, the development of materials that could be applied to different vaccine platforms regardless of product, such as the quality of mRNA, would negate the need for product-specific materials and thus might have broad utility.

Inevitably there are limited stability data on the control materials developed by and in use by the manufacturers. The NCL has access to these reference materials and thus adds an independent assessment of their performance to ensure consistency of vaccine production, until such time as this can be supported by independently calibrated reference materials that are formally established by standardisation bodies such as WHO and Pharmacopoeia. The NCL trends its own data to monitor the stability and performance of reference/control materials and vaccine batches over time, as well as the critical product characteristics from the manufacturer’s testing as evidenced through the LRP. For control or reference materials, this typically involves the recording of the values obtained, from each assay performed, reviewing individual or trending changes that could prompt evaluation of the material stability, and assay or analyst performance, for example. Statistical analysis confirms the presence of a trend. Trending data on parameters from each vaccine batch – such as potency or RNA integrity – could highlight stability or production consistency changes.

The WHO is developing written standards within its Technical Report Series relevant to the development, production, and evaluation of COVID-19 vaccines^11^; it has also signposted interested parties to existing guidelines that may be applicable to manufacturers and regulatory bodies.

### Additional benefits of NCL activity

The scientific and analytical expertise available within National Control Laboratories such as NIBSC enables the development and validation of new or improved analytical methods. The collaboration between NIBSC and GSK Vaccines for the development of an improved safety test for Bexsero (Meningococcal serogroup B vaccine) is an excellent example of how shared expertise can facilitate the rapid development of an improved method that is better suited to control a critical quality attribute of a vaccine^12–14^. Experts at NIBSC also provided scientific and regulatory support for the development of a group A meningococcal vaccine for sub-Saharan Africa^15^, the introduction of which has led to substantial reductions in group A meningococcal meningitis in this region^16^.

NCLs employ approaches such as proficiency testing schemes involving multiple laboratories to evaluate their own performance and harmonise test protocols. In time, these will be a valuable opportunity in defining standardised protocols for independent COVID-19 vaccine testing.

COVID-19 vaccines may be updated to meet public health demands arising from new SARS-CoV-2 variants. In essence, an update creates a new vaccine that may have properties different from those of the original, however subtle. There is a precedent for this in the seasonal amendments to the composition of influenza vaccines^17^. As one of four global WHO Essential Regulatory Laboratories (ERL), NIBSC is involved in a collaborative programme to develop and evaluate key reagents to support the manufacture and independent control testing of seasonal influenza vaccines for both Northern and Southern hemispheres. The expertise available within NCLs such as NIBSC will ensure that they play an important role in providing continued assurance for COVID-19 vaccine products and modified or updated versions of them.

## Conclusion

The independent testing of COVID-19 vaccines prior to their availability to the market adds a critical and impartial quality and safety assurance step. With COVID-19 vaccination programmes as a current exemplar, vaccines are evaluated in clinical trials involving thousands of participants and then rolled out to millions of healthy individuals. The pandemic has called for compressed timelines and highlighted the potential of novel platforms such as mRNA vaccines for infectious diseases. Trust in the vaccines by both the public receiving vaccinations and authorities responsible for the vaccination programmes and post-marketing surveillance are supported through testing which is independent of the manufacturers. The physical testing of samples of the vaccine batches by independent NCLs, as opposed to review only of manufacturers’ data, strengthens the confidence in vaccines and novel technology platforms.

## Data Availability

Data sharing is not applicable to this article as no datasets were generated or analysed for this Perspective.
